# Supplementary material for: Pyruvate Dehydrogenase Kinase 1 inhibition mediated oxidative phosphorylation enhancement in cartilage promotes osteoarthritis progression
Source: BMC Musculoskelet Disord. 2023 Jul 20;24:597. doi: 10.1186/s12891-023-06585-6 (PMC10357736; doi:10.1186/s12891-023-06585-6)
Supplement: Supplementary file 1 — Additional file 1. [file 12891_2023_6585_MOESM1_ESM.zip › KEGG.Gsea.1653622432464/KEGG_RENIN_ANGIOTENSIN_SYSTEM.html]

Details for gene set KEGG\_RENIN\_ANGIOTENSIN\_SYSTEM[GSEA]

|  || Dataset | 55457\_collapsed\_to\_symbols.cls.cls #OA\_versus\_normal.cls.cls #OA\_versus\_normal\_repos |
| Phenotype | cls.cls#OA\_versus\_normal\_repos |
| Upregulated in class | normal |
| GeneSet | KEGG\_RENIN\_ANGIOTENSIN\_SYSTEM |
| Enrichment Score (ES) | -0.18393236 |
| Normalized Enrichment Score (NES) | -0.5347805 |
| Nominal p-value | 0.9825175 |
| FDR q-value | 0.9948576 |
| FWER p-Value | 1.0 |
Table: GSEA Results Summary

  

Fig 1: Enrichment plot: KEGG\_RENIN\_ANGIOTENSIN\_SYSTEM      
 Profile of the Running ES Score & Positions of GeneSet Members on the Rank Ordered List

  

| SYMBOL | TITLE | RANK IN GENE LIST | RANK METRIC SCORE | RUNNING ES | CORE ENRICHMENT || 1 | CMA1 | chymase 1 [Source:HGNC Symbol;Acc:HGNC:2097] | 1244 | 0.468 | 0.0124 | Yes |
| 2 | CPA3 | carboxypeptidase A3 [Source:HGNC Symbol;Acc:HGNC:2298] | 1259 | 0.465 | 0.1197 | Yes |
| 3 | CTSA | cathepsin A [Source:HGNC Symbol;Acc:HGNC:9251] | 2014 | 0.360 | 0.1450 | Yes |
| 4 | CTSG | cathepsin G [Source:HGNC Symbol;Acc:HGNC:2532] | 2457 | 0.307 | 0.1823 | Yes |
| 5 | MAS1 | "MAS1 proto-oncogene, G protein-coupled receptor [Source:HGNC Symbol;Acc:HGNC:6899]" | 3629 | 0.191 | 0.1359 | No |
| 6 | AGT | angiotensinogen [Source:HGNC Symbol;Acc:HGNC:333] | 4407 | 0.120 | 0.1037 | No |
| 7 | THOP1 | thimet oligopeptidase 1 [Source:HGNC Symbol;Acc:HGNC:11793] | 5334 | 0.045 | 0.0423 | No |
| 8 | AGTR1 | angiotensin II receptor type 1 [Source:HGNC Symbol;Acc:HGNC:336] | 6122 | -0.014 | -0.0155 | No |
| 9 | ACE2 | angiotensin converting enzyme 2 [Source:HGNC Symbol;Acc:HGNC:13557] | 6951 | -0.076 | -0.0622 | No |
| 10 | ENPEP | glutamyl aminopeptidase [Source:HGNC Symbol;Acc:HGNC:3355] | 8521 | -0.195 | -0.1384 | No |
| 11 | AGTR2 | angiotensin II receptor type 2 [Source:HGNC Symbol;Acc:HGNC:338] | 9009 | -0.232 | -0.1221 | No |
| 12 | MME | membrane metalloendopeptidase [Source:HGNC Symbol;Acc:HGNC:7154] | 9287 | -0.255 | -0.0843 | No |
| 13 | ACE | angiotensin I converting enzyme [Source:HGNC Symbol;Acc:HGNC:2707] | 9720 | -0.294 | -0.0494 | No |
| 14 | LNPEP | leucyl and cystinyl aminopeptidase [Source:HGNC Symbol;Acc:HGNC:6656] | 10453 | -0.367 | -0.0206 | No |
| 15 | REN | renin [Source:HGNC Symbol;Acc:HGNC:9958] | 10889 | -0.416 | 0.0424 | No |
| 16 | ANPEP | "alanyl aminopeptidase, membrane [Source:HGNC Symbol;Acc:HGNC:500]" | 11404 | -0.487 | 0.1161 | No |
Table: GSEA details [plain text format]

  

Fig 2: KEGG\_RENIN\_ANGIOTENSIN\_SYSTEM      
 Blue-Pink O' Gram in the Space of the Analyzed GeneSet

  

Fig 3: KEGG\_RENIN\_ANGIOTENSIN\_SYSTEM: Random ES distribution      
 Gene set null distribution of ES for **KEGG\_RENIN\_ANGIOTENSIN\_SYSTEM**
